# Reactivation of Atrium Genes Is a Primer for Heart Infarction or Regeneration

**DOI:** 10.3389/fcvm.2020.575275

**Published:** 2020-11-05

**Authors:** Yisong Zhen

**Affiliations:** State Key Laboratory of Cardiovascular Disease, National Center for Cardiovascular Diseases, Fuwai Hospital, Chinese Academy of Medical Sciences and Peking Union Medical College, Beijing, China

**Keywords:** cardiac regeneration, myocardial infarction, bioinformatics analysis, cardiac atrium genes, proliferation signal

## Abstract

The inability of the adult heart to repair or regenerate is manifested in prevalent morbidity and mortality related to myocardial infarction and heart failure. However, the cue to the reactivation of cardiomyocyte proliferation in the adult remains largely unknown. In the present study, three independent datasets were explored using bioinformatics analysis methods to solve the problem. Our results revealed that atrium genes were upregulated in response to the injury, which indicates the possible cell type withdraw and reinitiation of proliferation capability. Our findings might provide an alternative viewpoint on the cardiomyocyte regeneration or myocardial infarction.

## Introduction

Cardiovascular diseases (CVDs) have long been the leading cause of global mortality and are a major contributor to reduced quality of life. To illustrate the molecular mechanism, basic research scientists used the concept of cardiac hypertrophy to describe a certain stage during the progression of various kinds of CVDs (1). They also designed experiments focusing on this phenotype to tackle drug targets and jigsaw the protein pathways. With the advance of translational medicine, the new perspective shifts to restore the capability of cardiomyocyte proliferation, which heralds the era of regenerative medicine. The simplified research theme suggests that the cardiomyocyte loss is the cause of CVD malfunction. Today, physician-scientists at the bench-side propose three strategies, including cell-based therapy (2, 3), bioengineering methods (4, 5), and synthetic organogenesis (6–8) to cure the diseases.

However, a major barrier against myocardial regeneration appears to be a cell-cycle arrest of adult mammalian cardiomyocytes. Two models explain why cardiomyocytes stop dividing. One theory is that mitosis of incessantly contractile cells is infeasible (9). Another theory is that neonatal heart regeneration in mammals is simply a remnant of developmental programs that were lost after birth (10, 11). On the other hand, accumulating evidence reveals another aspect of the story. The first is that the enforced expression cell-cycle kinase can restore the cardiac regeneration capacity (12). The second is the finding that the hypoxic cardiomyocyte is a rare population of proliferative cardiomyocyte responsible for the cellular turnover during the adult stage (13). The entry point to the proliferative phase remains unknown, and researchers around the world now use a wide variety of animal models (14) to tackle the problem.

In the zebrafish model, differentiated atrial cardiomyocytes can transdifferentiate into ventricular cardiomyocytes to contribute to cardiac ventricular regeneration (15). Another group found that a subpopulation of cardiomyocytes that transiently express atrial myosin heavy chain (*amhc*) contributes to specific regions of the ventricle (16). These findings suggest an unappreciated level of plasticity during chamber formation. Therefore, we hypothesize that there might be a kind of heart atrium progenitor cell that exists in the mammalian system. The progenitor cell will hence play a role in heart regeneration in response to injury stimuli. We designed the following study using public data to explore the possibility and revealed that heart regeneration came into being along with the emergence of the so-called atriumization, the upregulation of atrium specific genes.

## Methods

### Data Sources

All three datasets used in the present study were downloaded from Gene Expression Omnibus (GEO). The dataset GSE1479 is a benchmark set for early cardiac development and includes seven developmental stages from stage 10.5 to stage 18.5. At stage 10.5, the middle part of the mouse embryo, which includes the heart, was subjected to expression analysis. From embryonic day 11.5 on, embryonic hearts were isolated and the ventricular from the atrial chambers were separated. The GSE775 dataset is a time-series experiment design intended to compare normal functioning left ventricles (lv) with infarcted (ilv) and non-infarcted left ventricles (nilv). The original data manual described in detail the tissue sampling protocols that they sectioned the heart to three parts including Ilv, lv, and Nilv. Ilv samples are taken from the mouse myocardial infarction zone. Nilv samples are taken from the region above the infarction, and the left ventricle (lv) samples mimic that region in a sham mouse. In the traditional experiment design, the lv samples are the control and used to capture the differentially expressed genes. Both GSE1479 and GSE775 were released by the CardioGenomics project (17). The GSE64403 was published by Boyer's lab on the subject of transcriptional reversion of cardiac myocyte fate during mammalian cardiac regeneration (18). Primary myocardial tissues sampled from neonatal mice and murine hearts undergoing post-injury regeneration were harvested according to the original description.

### Collation of Atrium-Specific Genes

Atrium (specific) genes are highly expressed in the heart atrium compared with these in the ventricle. Therefore, atrium genes play important roles in chamber physiological function or morphogenesis during heart development. GSE1479 is a benchmark set for early cardiac development. From embryonic day 11.5 on, embryonic hearts were isolated and separated to the ventricular from the atrial chambers. In the bioinformatic curation procedure, the microarray data from respective chambers were pooled together to perform gene differential expression analysis using limma package (19). The expression value contrast above 1 (logFC > 1) and *p*-value above 0.001 were chosen as the complete chamber specific genes. After filtering, the hierarchical clustering was used to separate the atrium and ventricle gene sets. Manual curation of chamber-specific genes was undertaken to construct an independent evaluation set. This step usually is to read the literature and garner the atrium genes, the expression pattern of which at least is supported by Northern blot analysis.

### Source Code Management

The source code of the present project was deposited in the GitHub [atriumization_source_code.R (20)]. The development process tried to follow the etiquette of the reproducible research (21).

### Data Analysis Tools

Data were analyzed using the R/Bioconductor packages. Picture and graph layout was generated by ggplot2 and cowplot packages. Differential gene expression analysis was conducted using limma if the data type is Affymetrix microarray, or DESeq2 if the data is high-throughput sequencing. Gene Set Enrichment Analysis (GSEA) is a computational method that determines whether an *a priori* defined set of genes shows statistically significant, concordant differences between two biological states (22). This approach was conducted using the ClusterProfiler package. DAVID analysis was called locally using RDAVIDWebService to maintain code cleanness (23, 24). Heatmap analysis was generated using the pheatmap package. Principle component analysis results were generated using the factoextra and FactoMineR packages. Most packages were downloaded from Bioconductor or CRAN (25).

## Results

### Atrium-Specific Genes Curated From the Public Dataset

**Figure 1:**
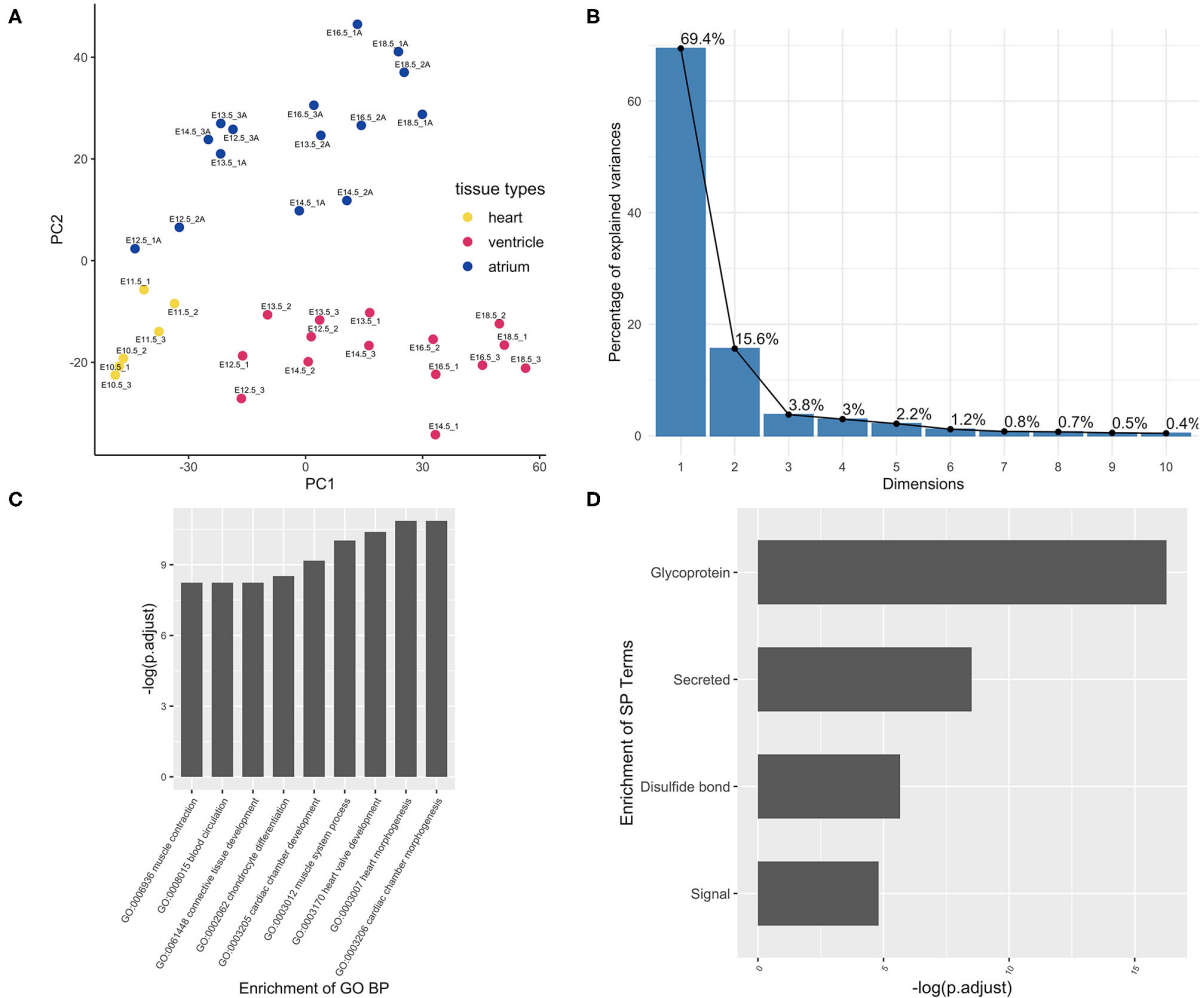
Curation of atrium-specific genes from the benchmark set GSE1479 (*n* = 36). The dataset is a microarray data of high quality generated by CardioGenomics. The data-mining methods were used to find the chamber-specific genes from the developmental stages. **(A)** The principal component analysis was performed to check the distribution of the atrium/ventricle groups in the experiment samples. Blue dots were samples from the atrium group. Yellow dots were samples from the whole heart group at the early stages. Red dots were samples from the ventricle group. These three kinds of tissue sample groups from different stages can be separated by the principal components PC1 and PC2. **(B)** After filtering the chamber-specific genes using the differential expression analysis and hierarchical clustering, a PCA procedure using the updated dataset was performed and the corresponding scree plot was generated. The new PC1 can explain nearly 70% variability of the data. **(C)** The set of atrium genes was analyzed using clusterProfiler and **(D)** DAVID pipeline, respectively. The enriched GO Biological Process (BP) annotation and the enriched term are consistent with our expectation, indicating that the chamber gene classification is of high accuracy.

Principle component analysis (PCA) was performed to check the data quality (GSE1479). The original experiment claimed to monitor changes in gene expression related to heart development and maturation. From embryonic day 11.5 on, the expression profiling by array also reported the gene expression, respectively, from the ventricular and atrial chambers. In the present PCA results, components PC1 and PC2 can perfectly separate these two events, cardiac maturation and chamber differentiation, although the maturation as an independent variable was partially collineared with chamber differentiation (Figure 1A). To minimize the cofounder of the maturation event, the chamber-specific samples at different developmental stages were pooled together and gene differential expression analysis was performed to curate the chamber-specific genes, and then hierarchical clustering was conducted to obtain these two groups. After the above filtering, the atrium- and ventricle-specific gene sets were generated accordingly. The atrium gene set included 189 genes each identified with a unique Entrez gene ID. The ventricle gene set included 99 genes, and the ratio of atrium and ventricle gene number is about 2:1 (Supplementary Table 1). The chamber-specific set was then used in the next round of PCA analysis, which resulted in the increased percentage of the PC1 component, and about 70% variability can be explained by the new PC1 (Figure 1B). In the atrium gene set, *sarcolipin* (*Sln*, gene ID: 66402) was included, which is a hallmark of heart atrium (26, 27). To further confirm these findings, a panorama view by the DAVID functional analysis was performed (Figures 1C,D). GO terms “cardiac chamber morphogenesis” and “heart morphogenesis” were enriched in the result. The term “secretion” was enriched in protein function using DAVID analysis, which was consistent with the previous observation that the atrium is a secretory organ (28).

### Expression of the Atrium Gene Set Was Rekindled in Myocardial Infarction

**Figure 2:**
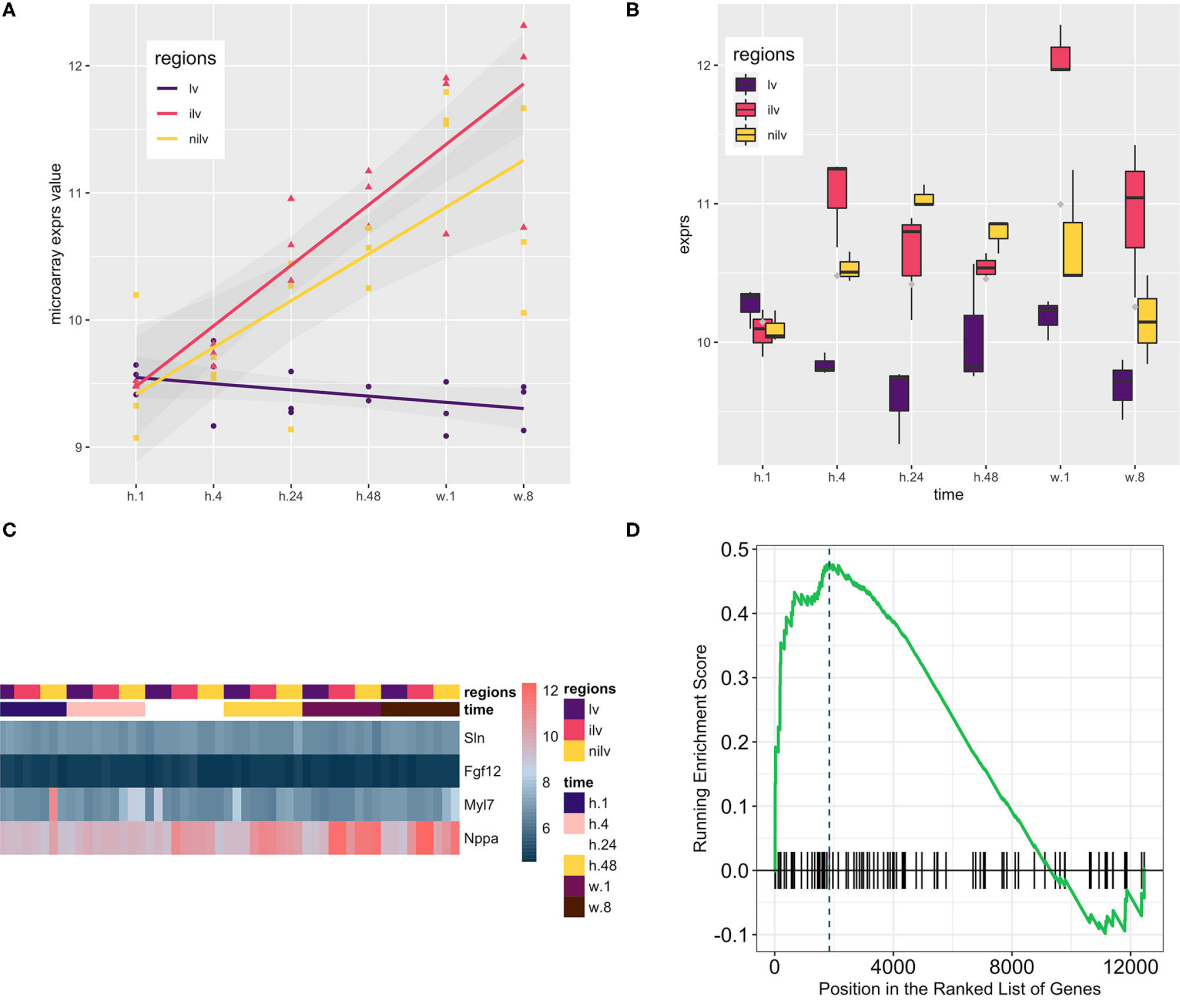
Using the atrium gene set in the myocardial infarction model (GSE775, *n* = 54) and the corresponding Gene Set Enrichment Analysis (GSEA) result. **(A)** *Natriuretic Peptide A* (*Nppa)* as the golden heart hypertrophy marker was assayed at the whole time points, and the regression line was generated to show the upregulation of *Nppa*. The upward of the regression line indicated that *Nppa* expression was positively associated with myocardial infarction procedure. **(B)** *Actin Alpha 2, Smooth Muscle* (*Acta2*) as the alternative marker was used to corroborate the previous finding. Comparison of infarcted left ventricles (iIv) or non-infarcted left ventricles (Nilv) with left ventricles (lv) is of statistical significance after multiple comparison adjustment by Bonferroni correction (data not shown). **(C)** A small group of manually curated atrium genes was displayed alongside with *Nppa*. The wave-like expression pattern of *Nppa* is consistent with the previous reports. However, the atrium genes were kept not fluctuating. The color scale bar at the left side of the heat map displays the raw log2-transformed expression value, ranging from 4.5 to 12.5. However, the raw value was not further scaled in a row or column direction. **(D)** The GSEA procedure was performed, and the result showed that the expression level of the atrium dataset at stage week 1 was increased significantly (*P* = 0.00501).

Dataset GSE775 was reanalyzed using the above-defined set for the atrium genes. This set includes spatiotemporal results by the microarray design. The original study included the time-serial samples, and the experiment also included three myocardial regions (17). Reactivation of *natriuretic peptide A* (*Nppa*) and *smooth muscle actin alpha 2* (*Acta2*) is the golden standard to assess the quality of the myocardial infarction model. The increased expression of both indicated that the mouse model is qualified for downstream analysis (Figures 2A,B). Expression of the atrium hallmark gene *Sln* is not statistically significant, however. The manual curated atrium genes plus *Nppa* were plotted using the heat map. Only the heart stress indicator, *Nppa*, was fluctuated with the time change. The GSEA analysis using the defined atrium gene set was then performed to assess the phenotype change. The result showed that the increased expression of atrium genes was of statistical significance (Figure 2D).

### Expression of the Atrium Gene Set Was Enlightened in Heart Regeneration

**Figure 3:**
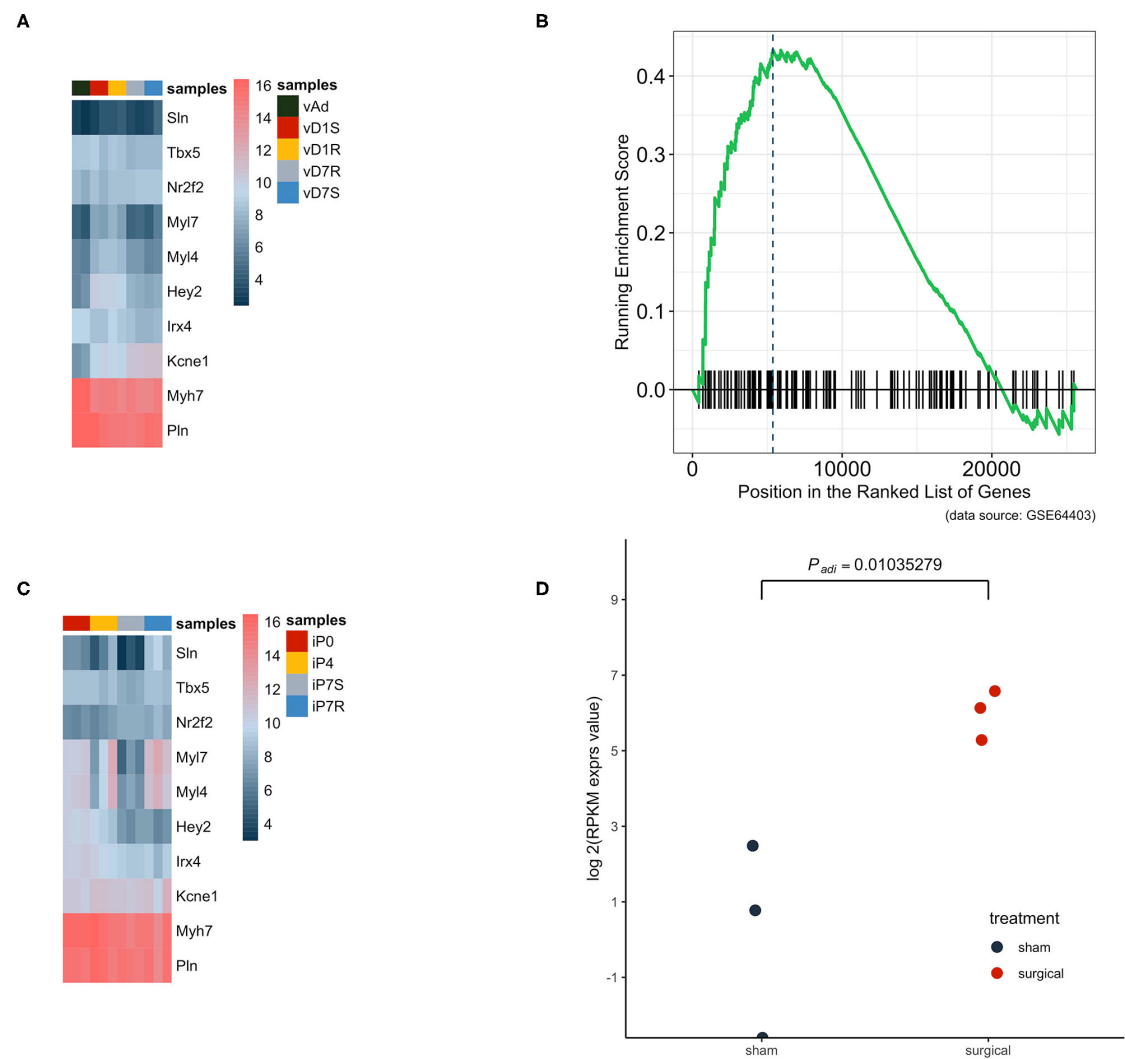
The atrium gene expression pattern in GSE775 was replicated in an independent dataset GSE64403. **(A)** In the heart ventricle tissues, the independent manually curated atrium genes were not significantly increased and the same were ventricle genes (*n* = 10). **(B)** The GSEA analysis result showed that the increased expression pattern of the atrium gene set at day 7 was of statistical significance (*P* = 0.0262, *n* = 4). **(C)** The same group of chamber genes was analyzed in the isolated heart cells, which did not include other lineage cells. The upregulated pattern in the heat map can be identified by observing the expression pattern of *Sln, Myl7*, and *Myl4* (*n* = 12). **(D)** The golden marker of the heart atrium, *Sln* was analyzed at day 7 and the expression level was compared between the surgical and sham groups. The upregulated *Sln* expression was statistically significant (nbinomTest from DESeq package, *P* < 0.05, *n* = 6).

Dataset GSE66403 was used as the biological and technique replicate to infer the phenotype transformation during heart regeneration (Figure 3). However, the new dataset is much more sensitive as the RNA-seq has a more accurate measurement. The chamber-specific gene set, which was manually curated from the published literature, was used as an independent set to deduce the phenotype change in the heat-map analysis. In the myocardial infarction dataset, the expression of atrium and ventricle genes was not altered. However, the GSEA analysis indicated that the expression of atrium genes was increased. The cell's physiological character was atrium-cell-like instead of ventricle-cell-like, while as an independent replicate in the heart regeneration dataset, the expression of the atrium gene markers in the isolated ventricle cells was increased and statistically significant. Particularly, the expression of *Sln* was significantly increased (*P* < 0.05, Figure 3D).

### RA Signaling Upstream of Patterning Was Activated

**Figure 4:**
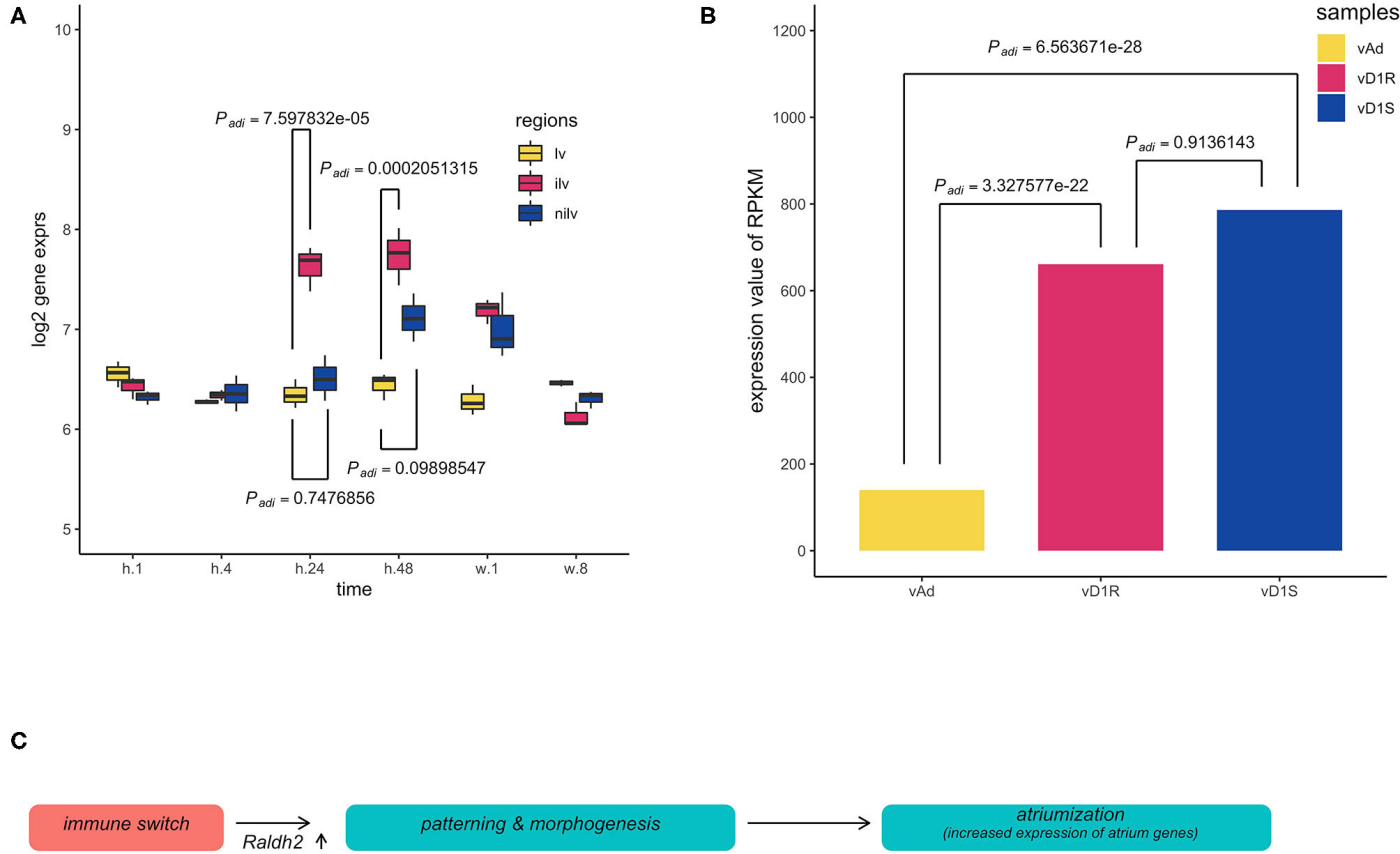
Multiple comparisons of aldehyde dehydrogenase family 1, subfamily A2 (*Aldh1a2*) expression among different groups in dataset GSE64403. **(A)** The *X*-axis stands for the sampling stages at 1 h, 8 h, 24 h, 48 h, 1 week, and 8 weeks in an operated mouse (*n* = 54). Lv stands for the left ventricle. Ilv stands for the infarcted and nilv is taken from the non-infarcted left ventricle. The results were obtained using Limma package, and *P-values* were adjusted using the Benjamini–Hochberg (BH) method. **(B)** vAd was the mouse sample from the region of the adult ventricular myocardium. vD1S was the mouse sample from the region of 1 day post-sham surgery ventricular myocardium. vD1R was the sample from the region of 1 day post-resection surgery ventricular myocardium. Expression of *Aldh1a2* from vD1R or vD1S was increased compared with that from vAd (*P*_*adj*_ < 0.001, *n* = 6). The *P*-values were calculated using the nbinomTest method in the DESeq package. The *P-value* adjustment was performed using the BH correction. **(C)** The graph summarized the inference of the present results, which hypothesized that the initial immune response stimulates the developmental patterning events during heart injury. Thus, the phenomenon of the so-called atriumization is the proxy for us to assess cardiac repair during heart regeneration or myocardial infarction.

To trace the upstream event, the retinoid acid pathway was explored in both datasets. Retinoic acid (RA) is a vitamin A metabolite that acts as a morphogen during body development. The retinoid pathway was monitored in the present analysis since it plays a role in cardiac chamber patterning. *Aldh1a2* (also named *Radlh2*), a component of the RA signaling, was activated in the epicardium during zebrafish heart regeneration (29). *Aldh1a2* was increased in these two datasets. The expression of *Aldh1a2* in the infarction site compared with the ventricle sham site or non-injury site was increased significantly at stages h.24 and h.48, respectively (Figure 4A). However, in the regeneration model, the expression of *Aldh1a2* was only significantly increased in the surgery heart compared with the normal heart (Figure 4B).

## Discussions

We rigorously defined the atrium gene set, including the ventricle gene set, using a benchmark dataset GSE1479. The myocardial infarction (GSE775) and heart regeneration (GSE64403) datasets were explored using data-mining methods. The results showed that the expression of atrium genes was increased in the harvested heart ventricle tissue. This observation can be explained by three interpretations. One possible mechanism is ventricular-to-atrial *trans*-differentiation, leading to the local increase of atrium cells. The second is atrium-like progenitor cell proliferation. The third may be the atrium cell migration from the heart atrium. We cannot exclude the other possibility. The discovery of this phenomenon is solely dependent on the reanalysis of these public data.

Verification of chamber-specific cells requires three pieces of evidence in the wet procedure. They include gene expression patterns, electrophysiological properties, and calcium sparks (30, 31). In the present study, a predefined atrium set was used in GSEA analysis, and an *in facto* atrium cell marker, *Sln*, was used to assess the atrium lineage. Adolfo J. de Bold proposed that, based upon the association of *Nppa* with the atrial-specific granules, the heart was an endocrine organ (32). We also found in the present unbiased bioinformatics approach that atrial cardiomyocytes displayed a secretory phenotype (Figure 1D). The heart was regarded as an endocrine organ 30 years ago, and secretome profiling was also the hallmark during cardiomyogenesis (33). The expression of *Nppa* was an early and specific marker for the differentiating working myocardium of the atria and ventricles of the developing heart (34). However, our statistical analysis did not support *Nppa* as the hallmark of the atrium phenotype. Instead, *Nppa* is an accurate marker in clinical investigation of cardiovascular diseases. Two separated enhancers govern independent response during development and disease (34). Instead, a recent study revealed that *Sln* is the true lineage marker of atrium cells and has a conserved expression pattern. Early work showed that *Sln* was upregulated during development and downregulated by cardiac hypertrophy (26). Thus, the expression of *Sln* is a perfect inner lineage control in our study. We found that the expression of *Sln* mRNA was detected selectively in the atria and not in myocardial infarction.

We hypothesized that the expression of atrium genes would be increased in response to cardiac damage (Figure 4C). Myocardial infarction (MI) is permanent damage to the heart muscle. The microarray data, GSE775, was then recruited to support our hypothesis. This mouse dataset is a time-series study intended to compare normal-functioning left ventricles (lv) with infarcted (ilv) and non-infarcted left ventricles (nilv). To double-check the quality of the dataset and analysis pipeline, the *Nppa* expression was assayed as it is the golden standard of cardiac diseases. Reactivation of ventricular *Nppa* expression is part of a conserved adaptive change of molecular phenotype in response to heart failure which serves both diagnostic and potential therapeutic options (35, 36). The expression levels of *Nppa* were regressed across the whole time points and upregulated in the ilv or nilv samples while that in lv sample (sham counterpart) was kept the same.

To further confirm the finding that the myocardial infarction model was successful, the expression of *Acta2* was checked as it is an alternative marker that was used to corroborate the finding (37). The upregulation of both *Nppa* and *Acta2* at the early stages indicates that the mouse model truly exhibits the disease progression. To monitor the expression level of the atrium genes, *Nppa* and manually curated atrium genes (26, 38, 39) were plotted in the same heat map. The wave-like expression pattern of *Nppa* (Figure 2C) was consistent with the previous analysis (Figure 2A). However, there was no change in expression of the atrium genes. The *Sln* expression is consistent with the previous finding that the expression in the heart ventricle was not upregulated during cardiac hypertrophic remodeling. The GSEA analysis was then undertaken to increase the power to detect the trend of expression direction. The result showed that the expression level of the atrium dataset was increased significantly.

To further confirm our finding that the expression of the atrium gene set was upregulated, the atrium gene expression pattern was verified in an independent dataset GSE64403, which utilized the deep-sequencing method and was a heart regeneration model. In the heart ventricle tissues, the atrium genes were not scientifically increased and the same were ventricle genes (Figure 3A). GSEA analysis was conducted and showed that the increased expression of atrium genes was of statistical significance. However, the harvested ventricle tissues included other cell lineages besides cardiac ventricle cells. To exclude this variable and identify the true source, the same group of chamber genes was analyzed in the isolated heart ventricle cells. The increased expression pattern can be identified in the authentic atrium markers, *Sln, Myl7*, and *Myl4* (Figure 3C). The golden marker of atrium lineage, *Sln*, was analyzed and compared between the surgical and sham groups of day 1. The upregulated *Sln* expression was statistically significant (*P* < 0.05). In the present study, we examined the effects of development and cardiac hypertrophy on the expression of *Sln* mRNA in the heart. We found that the expression of *Sln* was detected selectively in the atria. Furthermore, *Sln* was upregulated during development and downregulated by cardiac hypertrophy (26).

To ask what the upstream event of atrium gene reactivation is, multiple comparisons of *Aldh1a2* expression between different groups in data set GSE64403 were performed. An early study in zebrafish revealed that the injured heart at the epicardium exhibited increased expression of *Radhl2* using mRNA probe hybridization. The *P*-values were adjusted using the nbinomTest method in the DESeq2 package. The vAd sample was from the region of adult mouse ventricular myocardium. The vD1S was from the region of 1-day post-sham surgery ventricular myocardium. The vD1R was from the region of 1-day post-sham post-resection surgery ventricular myocardium. The expression of *Aldh1a2* from vD1R or vD1S was increased compared with that from vAd (*P*_*adj*_ < 0.001). *Hox* genes and retinoid receptors were also checked, and they were upregulated at different stages. These results indicate that at the earliest stage of myocardial infarction or heart regeneration, the immune response elicited the patterning process to be reactivated through the retinoid acid signaling pathway, which mirrors the developmental progress in cardiac development.

Numerous studies proposed that heart regeneration was reactivation of the development pathways (10). Nevertheless, this conclusion is not based upon the quantitative data. We here used the bioinformatic analysis to infer that the ventricle–atrium *trans*-differentiation might occur during the heart regeneration or myocardial infarction in the mouse model due to the reactivation of the body patterning event.

## Conclusions

The present approach using the public datasets and data-mining methods indicates that the increased expression of atrium-specific genes is the hallmark of local response to heart injury. The so-called atriumization then reveals possible phenotype transformation during heart regeneration. This kind of gene expression upregulation suggests that the heart ventricle cells prefer withdrawing their default physiological character and might be guided by the morphogen signal to turn into atrium-like cells. Here, the regression to the atrium cell in the damaged heart ventricle region might be the first step in response to local immune cue and possibly the phenomenon of atavism. In this sense, the atrium cardiac chamber is the default evolutionary appearance order (40). The cell-level reprogramming might be the fundamental mechanism of priming heart regeneration. Impediment of the process probably results in cardiomyocyte loss, leading to myocardial infarction or regeneration failure. Our work is only based upon the public data and needs to be addressed by wet verification. The preliminary results will shed light on the future investigation on regeneration medicine, however.

## Data Availability Statement

Publicly available datasets were analyzed in this study. This data can be found here: GSE775; GSE1479; GSE64403.

## Author Contributions

YZ is the sole author of the present work, which means that he made the conception design, acquisition, analysis, or interpretation of data for the work. He drafted the work and revised it critically for important intellectual content. He was the author that takes the responsibility for the final approval of the version to be published. He is also accountable for all aspects of the work in ensuring that questions related to the accuracy or integrity of any part of the work are appropriately investigated and resolved. All authors contributed to the article and approved the submitted version.

## Conflict of Interest

The author declares that the research was conducted in the absence of any commercial or financial relationships that could be construed as a potential conflict of interest.
